# Using a retrospective pretest instead of a conventional pretest is replacing biases: a qualitative study of cognitive processes underlying responses to thentest items

**DOI:** 10.1007/s11136-015-1175-4

**Published:** 2015-11-16

**Authors:** Elsbeth F. Taminiau-Bloem, Carolyn E. Schwartz, Florence J. van Zuuren, Margot A. Koeneman, Mechteld R. M. Visser, Carol Tishelman, Caro C. E. Koning, Mirjam A. G. Sprangers

**Affiliations:** Department of Medical Psychology, Academic Medical Center, University of Amsterdam, Amsterdam, The Netherlands; DeltaQuest Foundation, Inc., Concord, MA USA; Departments of Medicine and Orthopaedic Surgery, Tufts University School of Medicine, Boston, MA USA; Department of Clinical Psychology, University of Amsterdam, Amsterdam, The Netherlands; Department of General Practice, Academic Medical Center, University of Amsterdam, Amsterdam, The Netherlands; Department of Learning, Informatics, Management and Ethics, Medical Management Center, Karolinska Institutet, Stockholm, Sweden; The Innovation Center, Karolinska University Hospital, Stockholm, Sweden; Department of Radiation Oncology, Academic Medical Center, University of Amsterdam, Amsterdam, The Netherlands

**Keywords:** Response shift, Quality of life, Cancer, Thentest, Patient-reported outcomes, Item level, Cognitive processes

## Abstract

**Background:**

The thentest design aims to detect and control for recalibration response shift. This design assumes (1) more consistency in the content of the cognitive processes underlying patients’ quality of life (QoL) between posttest and thentest assessments than between posttest and pretest assessments; and (2) consistency in the time frame and description of functioning referenced at pretest and thentest. Our objective is to utilize cognitive interviewing to qualitatively examine both assumptions.

**Methods:**

We conducted think-aloud interviews with 24 patients with cancer prior to and after radiotherapy to elicit cognitive processes underlying their assessment of seven EORTC QLQ-C30 items at pretest, posttest and thentest. We used an analytic scheme based on the cognitive process models of Tourangeau et al. and Rapkin and Schwartz that yielded five cognitive processes. We subsequently used this input for quantitative analysis of count data.

**Results:**

Contrary to expectation, the number of dissimilar cognitive processes between posttest and thentest was generally larger than between pretest and posttest across patients. Further, patients considered a range of time frames when answering the thentest questions. Moreover, patients’ description at the thentest of their pretest functioning was often not similar to that which was noted at pretest. Items referring to trouble taking a short walk, overall health and QoL were most often violating the assumptions.

**Conclusions:**

Both assumptions underlying the thentest design appear not to be supported by the patients’ cognitive processes. Replacing the conventional pretest–posttest design with the thentest design may simply be replacing one set of biases with another.

**Electronic supplementary material:**

The online version of this article (doi:10.1007/s11136-015-1175-4) contains supplementary material, which is available to authorized users.

## Background

Change in patients’ quality of life (QoL) is most commonly assessed by means of the prospective baseline and follow-up design, also known as the pretest–posttest design [1–5]. However, response shift may occur in the interim, which is defined as a change in internal standards (recalibration), values (reprioritization) and/or the concept of QoL (reconceptualization) as a result of health changes [6]. Response shift may pose a serious threat to the pretest–posttest design, since it may render QoL assessments over time incomparable [7–10].

The retrospective pretest–posttest design is a commonly used approach to detect and control for recalibration response shift when measuring change in QoL [11–14]. The retrospective pretest or thentest extends the pretest–posttest design with a retrospective evaluation of an earlier assessment. Most times, respondents complete the conventional posttest assessment and are subsequently asked to complete the same questions again but with the instruction to provide a renewed judgment of their pretest functioning [15]. The first assumption of this design is that by taking posttest and thentest in close proximity, the content of respondents’ underlying cognitive processes will be consistent between posttest and thentest. Consequently, comparison of posttest and thentest scores would eliminate treatment-induced response shift and provide an unconfounded assessment of the treatment effect. In addition, the mean change in scores from pretest to thentest would provide an indication of the magnitude and direction of recalibration response shift [16]. The second assumption is that patients are able to accurately recall their pretest functioning when completing the thentest, and hence we expect consistency in the time frame and description of functioning referenced at pretest and thentest. However, the thentest has been criticized for its susceptibility to a number of biases, including memory distortion of pretest functioning, social desirability responding and the use of implicit theories of change [15, 17]. Interestingly, none of these assumptions has been tested qualitatively, at the level of the cognitive processes.

To the best of our knowledge, only Westerman et al. [18, 19] have qualitatively examined response strategies underlying QoL assessment using a thentest design. In their study, 23 patients with small cell lung cancer participated in cognitive think-aloud interviews at four time points during a treatment trajectory with chemotherapy. In presenting their results, Westerman and colleagues focussed on patients’ response strategies in the prospective measurement of QoL over time (i.e., comparison of pretest and posttest scores), rather than on the cognitive processes used in answering thentest items. However, they do mention that the interview transcripts indicate that patients have difficulty recalling the previous measurement point and/or their prior functioning when responding to the thentest items [18].

To examine the cognitive processes underlying the responses of patients with cancer to QoL (thentest) items, we have developed an analytic scheme [20] based on the frameworks of Tourangeau et al. [21] and Rapkin and Schwartz [22]. Combined, these models distinguish five cognitive processes underlying QoL assessments: (1) induction of a frame of reference, (2) recall of relevant information (i.e., experience sampling), (3) use of standards of comparison against which the retrieved information is judged, (4) use of an algorithm to prioritize and combine the retrieved information and (5) reporting and response selection, according to which the respondent may edit the initial response and subsequently maps the judgment onto the appropriate response category (see [1]). According to Rapkin and Schwartz [22], change in standards of comparison is indicative of recalibration response shift.

In a prior study, we used this analytic scheme to examine the content of the cognitive processes underlying patients’ responses to QoL items in a pretest–posttest design to study the assumption of consistency in the content of respondents’ cognitive processes over time. Our results showed that the content of all cognitive processes changed over time [1].

The present study builds on these prior results by examining whether the content of each distinct cognitive process underlying patients’ responses remains similar or changes between posttest and thentest. Since the thentest design is assumed to control for inconsistencies in respondents’ QoL assessment between pretest and posttest, we expect more congruence in the content of the cognitive processes underlying responses to the posttest and thentest than to the pretest and posttest. To comprehensively address both assumptions underlying the thentest design, we will also examine whether patients accurately recall their pretest functioning when completing thentest items. Finally, we will examine these assumptions at the individual patient and item level.

## Methods

This study continues work from our prior research project where pretest and posttest assessments (*n* = 50) [1] were either extended with a thentest (current study) or with transition questions [23]. In the current study, pretest and posttest data will only be used from the subjects who also provided thentest data. We refer to our prior publication for details on the Materials and methods [1] and here only provide a brief summary of the methods previously used, but expand on information unique for the present study.

### Participants

To include a heterogeneous sample with variation in cognitive processes used in arriving at responses to QoL (thentest) items, we purposefully selected patients with cancer with respect to gender, age and tumor site. All patients were undergoing radiotherapy at the Department of Radiation Oncology at the Academic Medical Center (AMC) in Amsterdam.

### Procedure

In accordance with designs commonly used in treatment evaluation, pretest assessments were administered prior to, and posttest and thentest assessments at the end of a salient health-related intervention. Pretest interviews took place on the same day the patient had an appointment for the CT-simulator to prepare the treatment or received the first radiation treatment. The posttest and thentest interviews were conducted on patients’ last day of radiotherapy. The interviews were conducted at the Department of Radiation Oncology of the AMC in 2008 by two researchers (ETB, MAK) not involved in the patient’s clinical care. All interviews were audio-recorded and transcribed verbatim.

To limit patient burden, we used seven items from the 30-item EORTC QLQ-C30 [24], a HRQoL instrument widely used in European cancer clinical trials [25]. Since we wanted to examine whether the cognitive processes would differ depending on the type of issue being assessed and whether it was general or specific, the items were selected such that they cover physical, psychological and social dimensions of HRQoL as well as global and specific content [20] (see Table 3, first column). All items employ a 1-week time frame. The thentest questions were adapted versions of these items, e.g., How would you rate your overall QoL in the week prior to the first interview?

We used Hak et al’s [26] Three-Step Test Interview that combines cognitive think-aloud interviewing and verbal probing techniques [27] at the pretest, posttest and thentest assessments to enable comparisons of patients’ cognitive processes. In the think-aloud interview, we asked the patients to read each question out loud and subsequently think out loud, describing their reasoning in assigning a score to the question. Immediately after the think-aloud response to each item, we attempted to elicit more information about participants’ cognitive processes using probes based on the cognitive process models of Tourangeau et al. [21] and Rapkin and Schwartz [22]. For example, in probing for the cognitive process comprehension/frame of reference we asked respondents what the target construct in the item (e.g., quality of life) means to them. To address the cognitive process reporting and response selection, we asked respondents why they chose the response category (e.g., “quite a bit”) and not an adjacent category (e.g., “a little”). For further details about the interview questions, we refer the reader to Ref. [1].

After completion of the posttest think-aloud interview, we introduced the thentest by jogging the patient’s memory about the time point at which s/he conducted the pretest think-aloud interview. As recommended, we tried to prompt recollection of the day and time of the pretest interview and provided cues to elicit patient’s memory [17, 28]. We then asked: “Please take a minute to think back to the first interview. The following questions concern the week immediately prior to this interview and the start of radiotherapy. At that time, you might have felt tense or sad, or maybe you didn’t feel tense or sad at all. You might have suffered from physical complaints, or maybe you didn’t suffer from physical complaints at all. Can you remember how you were feeling at that time?” Subsequently, we asked the patients to provide a new judgment about their QoL during the week prior to the pretest think-aloud interview. We emphasized that we were not asking patients to recall their pretest *response*, but rather to provide a renewed *judgment*. We used non-leading probes for the thentest items, such as “You just referred to your functioning prior to the start of radiotherapy, could you explain to me how you tried to recall this period?”

### Data analysis

**Fig. 1:**
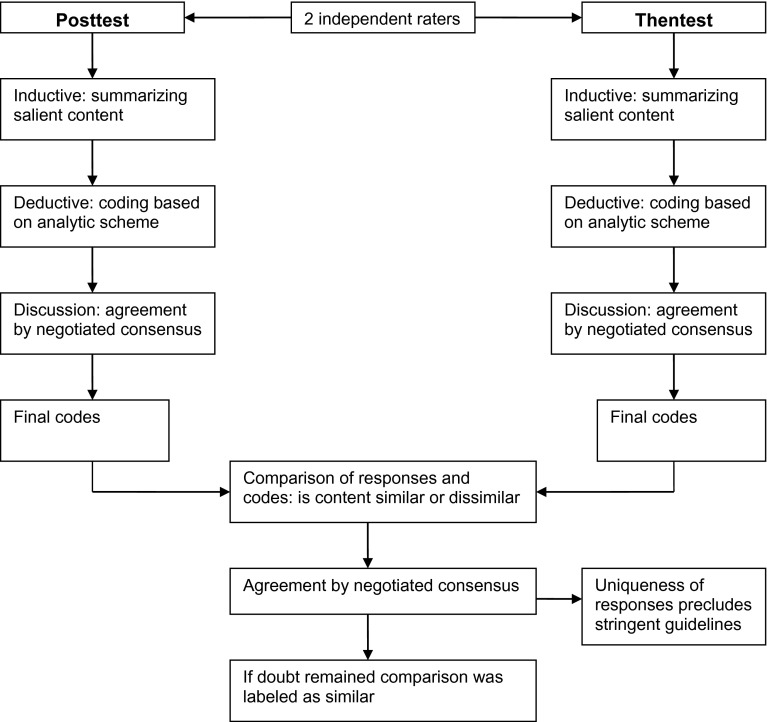
Flowchart of the data analysis process: posttest and thentest. *Note* the same process was used for analyzing the pretest and comparing the posttest with the pretest

Qualitative content analysis of all interviews was independently carried out by two researchers (ETB, MK) using MAXqda software [29]. The process of data analysis is graphically presented in Fig. 1. All responses of each assessment were first analyzed inductively by summarizing the salient content. All responses were then analyzed deductively using the same qualitative analytic scheme [20] (based on the cognitive process models of Tourangeau et al. [21] and Rapkin and Schwartz [22]) for the coding of patients’ cognitive processes as we used in our prior study [1]. The two researchers independently assigned codes related to the content of the underlying cognitive processes to all items of the pretest, posttest and thentest interviews of each patient. They discussed their findings and, in case of differences, achieved agreement about the assigned codes through negotiated consensus [30]. Once agreement was established, the assumption of consistency in the content of the cognitive processes underlying QoL appraisal between posttest and thentest (assumption 1) was examined. To that end, the researchers independently compared the content of each cognitive process at posttest and thentest to determine whether it was similar or had changed (see [1] for an illustration of the use of our analytic scheme). This dichotomy (similar vs. dissimilar) was input for quantitative analyses to examine whether the number of disparities was larger between the pretest and posttest than between the thentest and posttest in general, and across patients and items.

The second assumption of accurate recall underlying the thentest was operationalized by examining whether the time frame employed and the description of pretest functioning provided in answering each thentest item were similar to those of the corresponding pretest item. To that end, we compared the content of the cognitive processes comprehension/frame of reference and retrieval/sampling strategy at pretest and thentest. The codes were used as input for quantitative analyses. Again, all findings were discussed and consensus was negotiated in case of differences. Throughout the period of data collection and analysis, all codes and subsequent analyses were also discussed with co-authors FvZ and MS. Field notes were taken of decisions made to ensure consistency in the analysis.

## Results

Of the 38 eligible patients approached, 12 choose not to participate explaining they considered it too burdensome to be cognitively interviewed prior to and at the end of radiotherapy. Twenty-six patients gave written informed consent. One patient was unable to participate in the posttest and thentest interviews due to severe health deterioration, and one patient could not be interviewed at the end of radiotherapy due to logistical problems. The median number of days between the pretest interview, and the posttest and thentest interviews was 46 days (Mean 44 days, SD 8.7, range 27–57). Table 1 depicts the characteristics of the 24 patients who completed all three interviews (median age 61 years, SD 9.7, range 46–82). Twenty patients completed all seven items for all three assessments, with an additional two patients providing interpretable data for six items, and another two patients for five items.

**Table 1:** Patient characteristics

	No. of patients
Gender	
Men	12
Women	12
Age (years)	
40–49	4
50–59	7
60–69	10
70–79	2
≥80	1
Tumor site	
Bladder	2
Breast	4
Colorectal	4
Esophageal	4
Gynecological	3
Lung	2
Prostate	5

## Assumption 1 of the thentest design: the number of dissimilar cognitive processes between posttest and thentest is smaller than between pretest and posttest

Table 2 provides examples of patient responses at the posttest and thentest for each cognitive process. Contrary to the assumption, the number of dissimilar cognitive processes between posttest and thentest was found to be larger than between pretest and posttest across these patients for: frame of reference (103 vs. 94); standards of comparison (83 vs. 70); combinatory algorithm (37 vs. 35); and response selection (77 vs. 65). The only exception was sampling strategy (101 vs. 118).

**Table 2:** Assumption 1: Example quotes of dissimilar cognitive processes between posttest and thentest per cognitive process

Cognitive process	Patient	Item	Response	Posttest	Thentest
Comprehension/frame of reference (i.e., induction of a frame of reference)	Female, 48 years, breast cancer	Were you tired?	Response	A little	A little
			Definition	Patient defined fatigue as “loss of energy”	Patient defined fatigue as: “being out of balance”
			Explanation	“I am a little tired, I do notice that I am out of form. I kept putting on my track-suit to go for a run. But I noticed that when I had run 10 min on end, I lost my energy whereas normally that isn’t troublesome for me at all”	“I was out of balance because I was feeling tense”
Retrieval/sampling strategy (i.e., recall of relevant information)	Male, 78 years, esophageal cancer	Has your physical condition or medical treatment interfered with your social activities?	Response	A little	Not at all
			Sampling from	Radiotherapeutic treatment	The period prior to cancer diagnosis
			Explanation	“You have to drive to the hospital and back home every day, which really is a burden. And during that time you can’t be in contact with other people”	“No, not at all. I felt healthy, I wasn’t sick at that time. I kept company with the neighbors and close friends as always”
Standards of comparison (i.e., use of standards of comparison against which the retrieved information is judged)	Female, 59 years, gynecological cancer	How would you rate your overall quality of life during the past week?	Response	Score: 3	Score: 7
			Comparison	Current QoL to QoL prior to cancer diagnosis and treatment	QoL after cancer diagnosis, but prior to treatment to current, posttest QoL
			Explanation	“I hope my life will reestablish the way it used to be, and that was pretty good. (…) Currently, I feel put out of action. The only thing you do is lie on the couch, you no longer take part in normal, daily life”	“My quality of life was fine at that time. The fact that I already was diagnosed with cancer played a part, but I was able to do everything I wanted back then. And that is very different compared to my current quality of life”
Judgment/Combinatory algorithm^a^ (i.e., use of an algorithm to prioritize and combine the retrieved information)	Male, 58 years, prostatic cancer	How would you rate your overall health during the past week?	Response	Score: 4	Score: 6
			Emphasis on	Negative samples	Positive samples
			Explanation	“I was able to carry out some nice activities, we visited some friends, my e-mail. But I am no longer able to do what I want to do, without considering whether or not the distance is too long or whether it will exhaust me. So for the past week, it wasn’t that good. I was very tired, a ‘4’”	“I felt fine, I wasn’t in pain”; “I was suffering from the after-effects from my stroke back then, but I experienced lots of positive impulses. (…) I felt fine, I wasn’t in pain. So it was good, a ‘6’”
Reporting and response selection^b^ (i.e., mapping the judgment onto the appropriate response category)	Male, 78 years, esophageal cancer	How would you rate your overall health during the past week?	Response	Score: 5	Score: 5
			Reference to	Older age	Score previously provided at the posttest
			Explanation	“In the past couple of weeks, my health hasn’t been how I would like it to have been. But I guess it can’t be excellent anyway considering my age”	“It [health] wasn’t excellent and it wasn’t very poor either. So I’ll stick with the ‘5’ I’ve just chosen”

^a^In responding to a QoL item, patients can retrieve positive samples (e.g., “I’m not tired at all, because I still sleep soundly”) and negative samples (e.g., “The fact that I have to come to the hospital every day really tires me”) [Male, 52 years, esophageal cancer (both quotes)]. If patients retrieve both positive and negative samples, they need to combine this information to arrive at an answer. In doing so, patients can either emphasize the positive or negative samples, or find a balance between both

^b^Patients arrived at their score in varying ways at both the posttest and thentest interviews. For example, patients employed editing processes aimed at mitigating their initial response at either the posttest or thentest assessment. To elucidate further, patients did not refer to a previously provided score when answering the posttest item. However, at thentest, patients arrived at their score by referring to the score previously provided to the same item at posttest, or at pretest, or by referring to their previously provided score to another thentest item

**Fig. 2:**
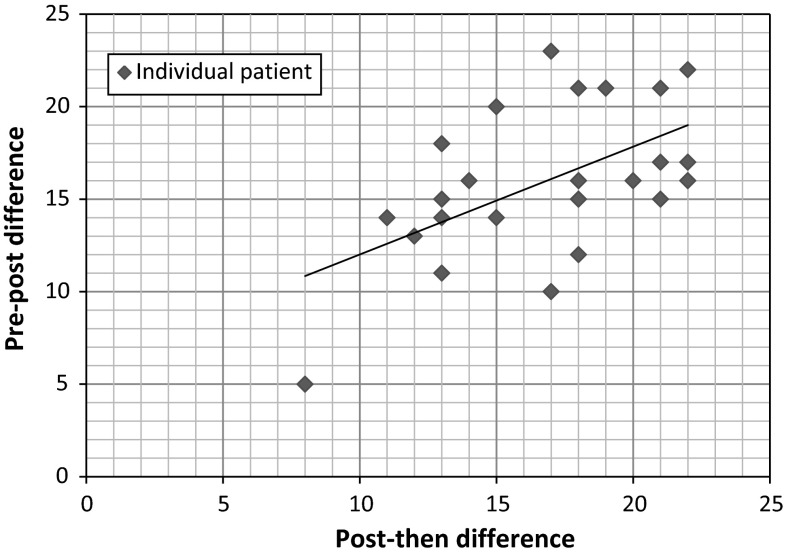
Scatterplot of pretest–posttest versus posttest–thentest disparity counts for all five cognitive processes combined (*regression line*)

At the patient level, there appears to be a linear trend such that patients with more discrepancies in cognitive processes between posttest and thentest also have more discrepancies in cognitive processes between pretest and posttest (see Fig. 2: scatterplot).

At the individual item level, items 1 (trouble taking a short walk) and 7 (overall QoL) induced the largest discrepancy in cognitive processes between the posttest–thentest versus pretest–posttest comparison (9 and 8 points, respectively, vs. 0–2 points for the remaining items; see Table 3).

**Table 3:** Assumption 1: number of cognitive processes across patients per individual item and Assumption 2: number of responses across patients per individual item

	Assumption 1		Assumption 2					
Items^a^	Pre–post: dissimilar processes	Post–then: dissimilar processes	Category 1: similar time frame; similar description (total of 60 responses)	Category 2: similar time frame; dissimilar description (total of 35 responses)	Category 3: dissimilar time frame; similar description (total of 10 responses)	Category 4: dissimilar time frame; dissimilar description (total of 57 responses)	Supportive of assumption 2 (category 1) (total of 60 responses)	Unsupportive of assumption 2 (categories 2-4) (total of 102 responses)
Item 1: Do you have any trouble taking a short walk outside of the house?	38^b^ (out of 92)^c^	47 (out of 92)	7	4	3	9	7	16
Item 2: Have you had pain?	53 (out of 104)	55 (out of 104)	11	3	0	10	11	13
Item 3: Were you tired?	52 (out of 97)	52 (out of 97)	8	5	1	9	8	15
Item 4: Did you worry?	46 (out of 103)	47 (out of 103)	11	4	0	9	11	13
Item 5: Has your physical condition or medical treatment interfered with your social activities?	54 (out of 98)	53 (out of 98)	8	7	2	6	8	15
Item 6: How would you rate your overall health during the past week?	78 (out of 102)	78 (out of 102)	7	4	4	7	7	15
Item 7: How would you rate your overall quality of life during the past week?	61 (out of 105)	69 (out of 105)	8	8	0	7	8	15

^a^Items 1–5 employ a four-point response scale; (1) not at all; (2) a little; (3) quite a bit; (4) very much. Items 6 and 7 employ a seven-point Likert scale, ranging from (1) very poor to (7) excellent

^b^Higher numbers mean worse performing item

^c^For each item a respondent can mention a maximum of five cognitive processes

## Assumption 2 of the thentest design: accurate recall of pretest functioning—comparing thentest and pretest

Example quotes for the second assumption are provided in Table 4. In comparing the thentest responses with the corresponding pretest responses, we found that the patients rarely used the 1-week time frame as instructed for the EORTC QLQ-C30 at both time points. Instead, a variety of different time periods were used. For the thentest, these include: “prior to cancer diagnosis and start of treatment,” “between cancer diagnosis and start of treatment,” “following other cancer treatment, but prior to radiotherapy,” or “other”, e.g., “since diagnosis of another illness.”

**Table 4:** Assumption 2: Example quotes per logical category

Category	Patient	Item	Response	Pretest	Thentest
Time frame: similar Description: similar	Female, 67 years, esophageal cancer	Have you had pain?	Response	Not at all	Not at all
			Time frame	Time prior to cancer diagnosis and treatment	Time prior to cancer diagnosis and treatment
			Description	“Naturally, I suffer from some small complaints. But that doesn’t bother me that much. (…) I have had these complaints for years now, and you get accustomed to it. If you want to grow old, that’s part of the deal”	“I haven’t had pain. Only those small complaints, which are part of your body getting older”
Time frame: similar Description: dissimilar	Male, 52 years, esophageal cancer	Have you had pain?	Response	Quite a bit	A little
			Time frame	Period between cancer diagnosis and start of treatment	Period between cancer diagnosis and start of treatment
			Description	“I have experienced pain when I received the diagnosis of having cancer. (…) When they tell you that, your entire world collapses completely. Some time after that message you recuperate, but I really suffered from pain psychologically”	“… Both mentally and physically”: “At that time, I suffered ‘a little’ pain because I had trouble swallowing. You are rebellious after the diagnosis, and you keep eating certain things that you can’t eat anymore. That makes you angry, resulting in suffering pain both mentally and physically”
Time frame: dissimilar Description: similar	Male, 64 years, colorectal cancer	Do you have any trouble taking a short walk outside of the house?	Response	Not at all	Not at all
			Time frame	Time prior to cancer diagnosis and treatment	Period between cancer diagnosis and start of treatment
			Description	“I haven’t got any trouble taking a walk outside. (…) I am capable of doing so, and I generally like it”	“After diagnosis, I walked as I have always done normally. Without limitations and without shortcomings”
Time frame: dissimilar Description: dissimilar	Female, 51 years, colorectal cancer	Did you worry?	Response	Very much	A little
			Time frame	Period prior to cancer diagnosis and start of treatment	Period between cancer diagnosis and start of treatment
			Description	“At first, I thought I had a hemorrhoid. But it got larger and larger until it grew out of my anus. At that stage I immediately realized I had to visit a physician. I worried a lot at that point, something needed to be done quickly”	“It’s too extreme to say I didn’t worry at all after receiving the diagnosis of having cancer. At that time I worried a little about possible metastases. You are in need of so much information”

The further comparison of the responses to the thentest items with the responses to the corresponding pretest items yielded four logical categories: (1) similarity in time frame and description of pretest functioning; (2) similarity in time frame and dissimilarity in description of pretest functioning; (3) dissimilarity in time frame and similarity in description of pretest functioning; and (4) dissimilarity in time frame and dissimilarity in description of pretest functioning. Whereas category 1 supports the assumption, the remaining three do not. The number of items across patients that was found to support the assumption was 60, whereas those in the three deviant categories totaled 102. The patients in this study thus more often used other time frames than those intended by the researchers, and provided a different description at the thentest than at the pretest. No clear pattern could be discerned regarding the four individual and two aggregated categories at the patient level. Table 3 depicts these data for each individual item. Items 1 (trouble taking a short walk) and 6 (overall health) were found to have the most discrepant time frames and descriptions between the pretest and thentest.

## Discussion

In this study, we examined the two assumptions inherent to the thentest design. Contrary to our expectations, the number of dissimilar cognitive processes between posttest and thentest was generally larger than between pretest and posttest across patients. Thus, these findings do not support the thentest design’s first assumption that the administration of the posttest and thentest in temporal proximity would induce a comparable content in cognitive processes. We also examined the thentest’s second assumption of accurate recall of pretest functioning. In comparison with their responses at pretest, patients more often provided a different description of their initial functioning and referred to a different time frame when responding to the thentest. In conclusion, both assumptions underlying the thentest design appear not to be supported by the cognitive processes described by the patients in this sample.

At the item level, items 1 (trouble taking a short walk), 6 (overall health) and 7 (overall QoL) were found to most notably violate the assumptions. Perhaps these items leave most room for personal interpretation (e.g., What is trouble? What is a short walk? What is QoL?). However, as we can imagine that other items (e.g., interference of medical treatment with social activities and worry) may also invite different views over time, it remains uncertain as to why some items seem to violate the assumptions more than others in this study. No discernable patterns were found at the patient level other than that patients with more discrepancies in the content of the cognitive processes between posttest and thentest also displayed more discrepancies between pretest and posttest.

According to Rapkin and Schwartz [22], change in the content of each of the cognitive processes constituting their QoL appraisal model is linked to one of the specific aspects of response shift, i.e., change in frame of reference is related to reconceptualization, change in sampling strategy and combinatory algorithm to reprioritization, and change in standards of comparison to recalibration. The thentest design is devised to detect and control for recalibration response shift [6], i.e., change in the respondent’s internal standards of measurement. Hence, in using the thentest, one would particularly expect similarity in the content of the cognitive process standards of comparison from posttest to thentest. However, our data do not reveal fewer disparities in content of standards of comparison between the posttest and thentest (83) than between the posttest and pretest (70).

Several studies have examined the accuracy of retrospective assessment of QoL as opposed to its prospective assessment. Some of these studies advocate the use of the thentest for treatment evaluation and consider it a more valid approach to measure change than standard, prospective measurement [9, 31–33]. Conversely, others raised validity concerns, suggesting that recall bias, social desirability and implicit theories of change may play a role in retrospective assessments [13, 34–36]. Our data suggest that this sample of patients may have used implicit theories of change. According to such a theory, patients infer what their pretest state must have been by extrapolating backwards from their current state [35]. In this study, we found instances where patients did not analyze their QoL at different time points but rather reconstructed their pretest functioning by using their current, posttest functioning as standard of comparison.

To increase our insight into patients’ response strategies, future studies are needed that examine how and why patients arrive at their answers. For example, studies could be designed in which patients are confronted with their pretest scores, after they have completed the thentest. Patients could subsequently be invited to comment their scores. Such interviews might provide valuable insight into patients’ own explanations of inconsistencies in the time frames employed and the descriptions of pretest functioning provided. In addition, to further elucidate actual response shift processes, it would be interesting to compare the content of all five cognitive processes between pretest and thentest in a more open and explanatory qualitative approach.

A number of limitations of this study warrant attention. First, given the small number of patients and the purposeful sampling strategy, we do not know how representative this patient group is and to what extent the characteristics of this particular sample may have affected the results. We do know, however, that patients who refused participation found the study too burdensome, which might indicate that the most severely ill patients were not included in this study’s sample. The interpretation of our findings needs thus be limited to the less severely ill [1]. Second, the heterogeneity of the seven EORTC QLQ-C30 items that we used for our interviews might have induced differences in the content of the cognitive processes used in answering the consecutive items. That is, questionnaire items addressing the same QoL domain might have resulted in more similarity in the content of the cognitive processes between posttest and thentest. However, standard QoL questionnaires, such as the EORTC QLQ-C30, are multi-dimensional by nature, thus including heterogeneous items by definition. We are also unsure whether a different selection of items would have resulted in different results. Third, as we chose to probe concurrently, the think-aloud process might have been influenced by the probing of the preceding item. However, in an earlier pilot test we found that probing retrospectively (after completion of all seven items) was unfeasible as respondents indicated to have forgotten their thought processes [20]. Finally, we cannot be sure that cognitive think-aloud interviews adequately capture patients’ cognitive processes [1].

We would also like to put forward a number of considerations when interpreting the results of this study. First, as is common in employing a thentest, we first have tried to revive patients’ memory about their pretest functioning. The thentest is most commonly administered in a written format. However, in the present study, we have orally instructed patients to think back to their pretest functioning. It is plausible that an oral instruction and subsequent think-aloud interview might have increased patients’ efforts to recall their pretest functioning. Consequently, in the context of conventional treatment evaluation, the results may show more deviation between pretest and thentest than was the case in the current study.

Second, our focus on the number of cognitive processes that has changed does not imply that a quantitative change is more important than a change in the content of cognitive processes. Dependent on the context, a change in one cognitive process may render responses over time more incompatible than changes in two or more cognitive processes. However, since the thentest design assumes that the content of each distinct cognitive process does not change between posttest and thentest, using the number of changed cognitive processes in this sample is informative. However, the absolute numbers should be interpreted with caution.

Third, the strict operationalizations of the two assumptions adopted here naturally affect the results. Both the pretest and thentest instruct patients to rate their QoL in the week prior to the pretest interview (i.e., after diagnosis, prior to treatment). For example, for the 59-year-old female with gynecological cancer (Table 2) the standard of comparison is at stake. This woman judges her posttest QoL in reference to her QoL *prior to* her cancer diagnosis and treatment, and her thentest QoL—referred to the period *after* diagnosis but prior to treatment—in reference to the QoL of life at the posttest. We concluded that she adopted a different standard of comparison at posttest than at thentest. However, one may argue that she is doing exactly what we want her to do at the thentest: judging her former QoL as compared to her current QoL. It should be noted that we adopted the same strict criteria for testing the first assumption for the conventional pretest–posttest design [1]. Therefore, our conclusions regarding the comparison of the two designs with respect to the first assumption are warranted.

Finally, our interpretation of recall bias merits attention as inconsistent time frames between pretest and thentest are not always indicative of recall bias per se. In the instances where patients did not adopt the requested time frame at the pretest, reference to the week prior to the pretest at the thentest (indication of accurate time frame) would still be counted as a discrepancy. Whereas the interpretation of recall bias in those instances is disputable [37], they still impair the validity of the thentest design.

In conclusion, the cognitive processes underlying patients’ responses to thentest items in this study appear not to be in line with the assumptions of (1) consistency in the content of the cognitive processes underlying responses to the posttest and thentest, and (2) accurate recall of pretest functioning. Rather, our data suggest that these patients select personally meaningful time frames and content when (retrospectively) assessing their QoL, which might deviate from the time frames considered relevant by researchers. The question arises whether the thentest is a suitable alternative in controlling for inconsistencies in respondents’ cognitive processes over time. However, as argued previously [38, 39], retrospective assessments, such as the thentest, are useful methods when the measurement goal is to examine change as experienced subjectively by the respondents. In interpreting thentest responses in the context of treatment evaluation, it is important to realize that patients provide assessments that are not necessarily based on the cognitive processes intended by researchers. Replacing the conventional pretest with a thentest may thus not resolve the bias underlying the prospective measurement of change in QoL, but rather may replace it with other biases, such as recall bias, social desirability, and implicit theories of change.

## Electronic supplementary material

Below is the link to the electronic supplementary material.

Supplementary material 1 (DOCX 30 kb)

## References

[1] Taminiau-Bloem EF, van Zuuren FJ, Koeneman MA, Rapkin BD, Visser MRM, Koning CCE, Sprangers MAG (2010). A ‘short walk’ is longer before radiotherapy than afterwards: A qualitative study questioning the baseline and follow-up design. Health and Quality of Life Outcomes.

[2] Morak MJ, Pek CJ, Kompanje EJ, Hop WC, Kazemier G, van Eijck CH (2010). Quality of life after adjuvant intra-arterial chemotherapy and radiotherapy versus surgery alone in resectable pancreatic and periampullary cancer: A prospective randomized controlled study. Cancer.

[3] Komblith AB, Huang HQ, Walker JL, Spirtos NM, Rotmensch J, Cella D (2009). Quality of life of patients with endometrial cancer undergoing laparoscopic international federation of gynecology and obstetrics staging compared with laparotomy: A Gynecologic Oncology Group study. Journal of Clinical Oncology.

[4] Groenvold M, Fayers PM, Petersen MA, Mouridsen HT (2006). Chemotherapy versus ovarian ablation as adjuvant therapy for breast cancer: Impact on health-related quality of life in a randomized trial. Breast Cancer Research.

[5] Weeks JC, Nelson H, Gelber S, Sargent D, Schroeder G, for the Clinical Outcomes of Surgical Therapy (COST) Study Group (2003). Short-term quality-of-life outcomes following laparoscopic-assisted colectomy vs open colectomy for colon cancer. JAMA.

[6] Sprangers MAG, Schwartz CE (1999). Integrating response shift into health-related quality-of-life research: A theoretical model. Social Science and Medicine.

[7] Schwartz CE, Feinberg RG, Jilinskaia E, Applegate JC (1999). An evaluation of a psychosocial intervention for survivors of childhood cancer: Paradoxical effects of response shift over time. Psycho-Oncology.

[8] Hagedoorn M, Sneeuw KCA, Aaronson NK (2002). Changes in physical functioning and quality of life in patients with cancer: Response shift and relative evaluation of one’s condition. Journal of Clinical Epidemiology.

[9] Visser MR, Oort FJ, Sprangers MA (2005). Methods to detect response shift in quality of life data: A convergent validity study. Quality of Life Research.

[10] Rees J, Clarke MG, Waldron D, O’Boyle C, Ewings P, MacDonagh RP (2005). The measurement of response shift in patients with advanced prostate cancer and their partners. Health and Quality of Life Outcomes.

[11] Andrykowski MA, Donovan KA, Jacobsen PB (2009). Magnitude and correlates of response shift in fatigue ratings in women undergoing adjuvant therapy for breast cancer. Journal of Pain and Symptom Management.

[12] Razmjou H, Yee A, Ford M, Finkelstein JA (2006). Response shift in outcome assessment in patients undergoing total knee arthroplasty. The Journal of Bone and Joint Surgery.

[13] Rees J, Waldron D, O’Boyle C, Ewings P, MacDonagh R (2003). Prospective vs retrospective assessment of lower urinary tract symptoms in patients with advanced prostate cancer: the effect of ‘response shift’. BJU International.

[14] Jansen SJ, Stiggelbout AM, Nooij MA, Noordijk EM, Kievit J (2000). Response shift in quality of life measurement in early-stage breast cancer patients undergoing radiotherapy. Quality of Life Research.

[15] Howard GS, Ralph KM, Gulanick NA, Maxwell SE, Nance SW, Gerber SK (1979). Internal invalidity in pretest-posttest self-report evaluations and a re-evaluation of retrospective pretests. Applied Psychological Measurement.

[16] Schwartz CE, Sprangers MAG (1999). Methodological approaches for assessing response shift in longitudinal health-related quality-of-life research. Social Science and Medicine.

[17] Schwartz CE, Sprangers MAG (2010). Guidelines for improving the stringency of response shift research using the thentest. Quality of Life Research.

[18] Westerman MJ, The AM, Sprangers MAG, Groen HJM, van der Wal G, Hak T (2007). Small-cell lung cancer patients are just ‘a little bit’ tired: Response shift and self-presentation in the measurement of fatigue. Quality of Life Research.

[19] Westerman MJ, Hak T, Sprangers MAG, Groen HJM, van der Wal G (2008). The AM: Listen to their answers! Response behaviour in the measurement of physical and role functioning. Quality of Life Research.

[20] Bloem EF, van Zuuren FJ, Koeneman MA, Rapkin BD, Visser MRM, Koning CCE, Sprangers MAG (2008). Clarifying quality of life assessment: Do theoretical models capture the underlying cognitive processes?. Quality of Life Research.

[21] Tourangeau R, Rips LJ, Rasinski K (2000). The psychology of survey response.

[22] Rapkin BD, Schwartz CE (2004). Toward a theoretical model of quality-of-life appraisal: Implications of findings from studies of response shift. Health and Quality of Life Outcomes.

[23] Taminiau-Bloem EF, Van Zuuren FJ, Visser MR, Tishelman C, Schwartz CE, Koeneman MA, Koning CC, Sprangers MA (2011). Opening the black box of cancer patients’ quality-of-life change assessments: A think-aloud study examining the cognitive processes underlying responses to transition items. Psychology and Health.

[24] Aaronson NK, Ahmedzai SA, Bergman B (1993). A quality of life instrument for use in international clinical trials in oncology. Journal of the National Cancer Institute.

[25] Garratt A, Schmidt L, Mackinstosh A, Fitzpatrick R (2002). Quality of life measurement: bibliographic study of patient assessed health outcome measures. British Medical Journal.

[26] Hak, T., van der Veer, K., Jansen, H. (2002). *The three*-*step test*-*interview (TSTI): An observational instrument for pre*-*testing self*-*completion questionnaires.* Paper for the International Conference on Questionnaire Development, Evaluation and Testing Methods (QDET). Charleston: South Carolina.

[27] Willis GB (2005). Cognitive interviewing: a tool for improving questionnaire design.

[28] Sprangers M, Hoogstraten J (1989). Pretesting effects in retrospective pretest-posttest designs. Journal of Applied Psychology.

[29] MAXqda (2004). [www.maxqda.com].

[30] Bowden JA, Bowden JA, Walsh E (1996). Phenomenographic research. Undertaking phenomenographic research: The Warburton symposium.

[31] Nieuwkerk PT, Tollenaar MS, Oort FJ, Sprangers MAG (2007). Are retrospective measures of change in quality of life more valid than prospective measures?. Medical Care.

[32] Ahmed S, Mayo NE, Wood-Dauphinee S, Hanley JA, Cohen SR (2004). Response shift influences estimates of change in health-related quality of life (HRQL) post-stroke. Journal of Clinical Epidemiology.

[33] Sprangers MAG, van Dam FSAM, Broersen J, Lodder L, Wever L, Visser M, Oosterveld P, Smets E (1999). Revealing response shift in longitudinal research on fatigue: The use of the thentest approach. Acta Oncologica.

[34] Hill LG, Betz DL (2005). Revisiting the retrospective pretest. American Journal of Evaluation.

[35] Norman G (2003). Hi! How are you? Response shift, implicit theories and differing epistemologies. Quality of Life Research.

[36] Eton DT (2010). Why we need response shift: An appeal to functionalism. Quality of Life Research.

[37] Blome C, Augustin M (2015). Measuring change in quality of life: Bias in prospective and retrospective evaluation. Value in Health.

[38] Hermann D (1995). Reporting current, past and change health status. What we know about distortion. Medical Care.

[39] Cella D, Hahn EA, Dineen K (2002). Meaningful change in cancer-specific quality of life scores: Differences between improvement and worsening. Quality of Life Research.

