# Supplementary material for: Using a retrospective pretest instead of a conventional pretest is replacing biases: a qualitative study of cognitive processes underlying responses to thentest items
Source: Qual Life Res. 2015 Nov 16;25:1327–37. doi: 10.1007/s11136-015-1175-4 (PMC4870298; doi:10.1007/s11136-015-1175-4)
Supplement: Supplementary file 1 — Supplementary material 1 (DOCX 30 kb) [file 11136_2015_1175_MOESM1_ESM.docx]

**Supplementary Material**

Supplementary Table 1: Assumption 1: Results at the individual patient level indicating dissimilarity in the cognitive processes, with the numbers reflecting the item numbers

Supplementary Table 2: Assumption 2: Results at the individual patient level, with the numbers reflecting the item numbers

**Supplementary Table 1: Assumption 1: Results at the individual patient level indicating dissimilarity in the cognitive processes, with the numbers reflecting the item numbers***

| **Patients** | **Comparison** | **Comprehension / frame of reference** | **Retrieval / sampling strategy** | **Standards of comparison** | **Judgment / combinatory algorithm**** | **Reporting and response selection** |
| --- | --- | --- | --- | --- | --- | --- |
|  |  |  |  |  |  |  |
| 1 | pre-post | 1 (trouble), 4, 5 (interference) | 1, 3, 4, 7 | 2, 6, 7 | 2, 6 (only items 2, 4, 5, 6 applicable for analysis) | 2, 3, 4, 5, 6 |
|  |  |  |  |  |  |  |
|  | post-then | 1 (trouble + walk), 3, 4, 5 (interference), 6, 7 | 4, 5 | 3, 4, 5, 6, 7 | 4, 5 (only items 2, 4, 5, 6 applicable for analysis) | 1, 2, 3, 4, 5, 6, 7 |
|  |  |  |  |  |  |  |
| 2 | pre-post | 1 (trouble), 3, 4, 5 (interference), 6, 7 | 2, 3, 5, 6, 7 | 2, 3, 4, 6, 7 | 1, 6 (only items 1, 6 applicable for analysis) | 5, 6, 7 |
|  |  |  |  |  |  |  |
|  | post-then | 3, 6, 7 | 3,6 | 1, 2, 3, 4, 6, 7 | 1 (only items 1, 6 applicable for analysis) | 1, 3, 4, 5, 6, 7 |
|  |  |  |  |  |  |  |
| 3 | pre-post | 1, 6 | 1, 2, 3, 5, 6, 7 | 2, 4, 6 | (no items applicable for analysis) | 2, 6, 7 |
|  |  |  |  |  |  |  |
|  | post-then | 6 | 1, 2, 3, 5, 6, 7 | 2, 6, 7 | (no items applicable for analysis) | 4, 6, 7 |
|  |  |  |  |  |  |  |
| 4 | pre-post | 1 (trouble + walk), 4, 5 (interference), 7 | 1, 3, 4, 5, 6, 7 |  | 4, 6 (only items 2, 3, 4, 6 applicable for analysis) | 4, 6, 7 |
|  |  |  |  |  |  |  |
|  | post-then | 1 (trouble), 2, 3, 4, 6 | 6, 7 | 1, 3, 4, 5, 7 | 3, 4, 6 (only items 2, 3, 4, 6 applicable for analysis) | 4, 6, 7 |
|  |  |  |  |  |  |  |
| 5 | pre-post | 3, 5, 6, 7 | 2, 3, 4, 5, 7 | 3, 6, 7 | 7 (only items 1, 6, 7 applicable for analysis) | 3, 5, 6 |
|  |  |  |  |  |  |  |
|  | post-then | 2, 4, 5 (interference), 6 | 2, 3, 5, 6, 7 | 2, 6, 7 | 6, 7 (only items 1, 6, 7 applicable for analysis) | 1, 4, 6, 7 |
|  |  |  |  |  |  |  |
| 6 | pre-post | 6 | 1, 2, 3, 6, 7 | 2, 6 | 6 (only items 3, 6, 7 applicable for analysis) | 3, 6, 7 |
|  |  |  |  |  |  |  |
|  | post-then | 6, 7 | 1, 2, 4, 6, 7 | 1, 2, 3, 6 | 3, 6, 7 (only items 3, 6, 7 applicable for analysis) | 2, 3, 4, 7 |
|  |  |  |  |  |  |  |

| 7 | pre-post | 2, 3, 6, 7 | 1, 2, 3, 4, 5, 6, 7 | 3, 5, 6, 7 | 2, 3, 5, 6 (only items 2, 3, 5, 6, 7 appl for analysis) | 2, 6 |
| --- | --- | --- | --- | --- | --- | --- |
|  |  |  |  |  |  |  |
|  | post-then | 2, 6, 7 | 1, 2, 4, 6 | 3, 5, 6, 7 | 2, 3, 5, 6, 7 (only items 2, 3, 5, 6, 7 appl for analysis) | 2, 4, 5, 6, 7 |
|  |  |  |  |  |  |  |
| 8 | pre-post | 1 (trouble), 2, 6 | 1, 2, 3, 6, 7 | 3, 4, 6 | 4, 6 (only items 1, 4, 5, 6 applicable for analysis) | 3, 4, 5 |
|  |  |  |  |  |  |  |
|  | post-then | 1 (trouble + walk), 2, 3, 6 | 1, 2, 6 | 1,3, 4, 6 | 1 (only items 1, 4, 5, 6 applicable for analysis) | 5, 6 |
|  |  |  |  |  |  |  |
| 9 | pre-post | 4, 5 (social activities), 6 | 4, 5, 6, 7 | 2, 3, 5, 6 | 5, 6 (only items 1, 3, 4, 5, 6, 7 appl for analysis) | 1, 2, 4, 6 |
|  |  |  |  |  |  |  |
|  | post-then | 1 (trouble), 2, 4, 5 (interf + soc act), 6 | 1, 2, 3, 4, 5 | 2, 3, 5, 6 | 1, 3, 4, 5, 6 (only items 1, 3, 4, 5, 6, 7 appl for analysis) | 4, 6 |
|  |  |  |  |  |  |  |
| 10 | pre-post | 3, 4, 5 (interference + social activities), 6, 7 | 4, 6, 7 | 1, 3, 4, 5, 6, 7 | (only items 2, 4, 7 applicable for analysis) | 3, 6 |
|  |  |  |  |  |  |  |
|  | post-then | 1 (trouble), 2, 3, 4, 5 (interference), 6, 7 | 1, 2, 3, 5, 6, 7 | 1, 4, 5, 6, 7 | 4 (only items 2, 4, 7 applicable for analysis) | 3, 6, 7 |
|  |  |  |  |  |  |  |
| 11 | pre-post | 5 (interference), 6, 7 | 2, 6, 7 | 2, 6, 7 | 5, 6, 7 (only items 5, 6, 7 applicable for analysis) | 2, 7 |
|  |  |  |  |  |  |  |
|  | post-then | 2, 5 (interference + soc activities), 6, 7 | 2, 5, 6 | 6, 7 | (only items 5, 6, 7 applicable for analysis) | 6, 7 |
|  |  |  |  |  |  |  |
| 12 | pre-post | 1 (trouble + walk), 2, 4, 5 (interference), 6 | 4, 6, 7 | 1, 2, 3, 4 | 2, 5 (only items 2, 3, 5, 7 applicable for analysis) | 3 |
|  |  |  |  |  |  |  |
|  | post-then | 1 (trouble), 2, 3, 4, 5 (interf + soc act), 7 | 1, 4, 5, 6 | 1, 2, 3, 6, 7 | 2, 3, 7 (only items 2, 3, 5, 7 applicable for analysis) | 1, 6, 7 |
|  |  |  |  |  |  |  |
| 13 | pre-post | 2, 3 | 1, 2, 3, 5, 6, 7 | 2, 3, 4, 5, 7 | (no items applicable for analysis) | 7 |
|  |  |  |  |  |  |  |
|  | post-then | 2, 3, 5 (interference + soc activities), 6 | 1, 2, 3 | 2, 3, 5, 6, 7 | (no items applicable for analysis) | 4, 6, 7 |
|  |  |  |  |  |  |  |
| 14 | pre-post | 2, 3, 5 (interference + social activities), 6, 7 | 1, 2, 3, 5, 6, 7 | 1, 2, 3, 5 | 6 (only items 2, 6, 7 applicable for analysis) | 2, 4, 6, 7 |
|  |  |  |  |  |  |  |
|  | post-then | 4, 6, 7 | 2, 4, 6, 7 | 1, 3, 5 | 6, 7 (only items 2, 6, 7 applicable for analysis) | 4, 6, 7 |
|  |  |  |  |  |  |  |
| 15 | pre-post | 1 (trouble), 3, 5 (interf + soc act), 6, 7 | 1, 2, 3, 5, 6, 7 | 1, 2, 5 | 2, 6 (only items 2, 6 applicable for analysis) |  |
|  |  |  |  |  |  |  |
|  | post-then | 1 (trouble), 3, 5 (interf + soc act), 6, 7 | 1, 2, 3, 4, 6, 7 | 1, 2, 5, 6 | 2, 6 (only items 2, 6 applicable for analysis) | 5, 6, 7 |
|  |  |  |  |  |  |  |
| 16 | pre-post | 2 | 2, 5 | 4 | (no items applicable for analysis) | 1 |
|  |  |  |  |  |  |  |
|  | post-then | 2 | 2, 5 | 2, 4, 5 | (no items applicable for analysis) | 1, 3 |
|  |  |  |  |  |  |  |
| 17 | pre-post | 2, 3, 4, 5 (interference + social activities), 6, 7 | 1, 2, 3, 4, 6, 7 | 2, 4, 6, 7 | 5 (only item 5 applicable for analysis) | 1, 3, 5, 7 |
|  |  |  |  |  |  |  |
|  | post-then | 2, 4, 5 (interference + soc activities), 7 | 1, 2, 3, 4, 5, 6, 7 | 1, 2, 5, 6 | (only item 5 applicable for analysis) | 1, 3, 6, 7 |
|  |  |  |  |  |  |  |
| 18 | pre-post | 1 (trouble), 2, 3, 5 (interference) | 1, 2, 3, 4, 5, 6, 7 |  | 5, 6, 7 (only items 4, 5, 6, 7 applicable for analysis) | 2, 4, 6, 7 |
|  |  |  |  |  |  |  |
|  | post-then | 1 (trouble), 2, 3, 5 (interference), 7 | 1, 2, 4, 5, 6 |  | 5 (only items 4, 5, 6, 7 applicable for analysis) | 2, 7 |
|  |  |  |  |  |  |  |
| 19 | pre-post | 1 (trouble + walk), 2, 3, 5 (interf + soc act), 6 | 1, 2, 3, 4, 5, 6, 7 | 2, 3, 7 | 2, 3 (only items 2, 3, 6, 7 applicable for analysis) | 1, 4, 5, 6, 7 |
|  |  |  |  |  |  |  |
|  | post-then | 1 (trouble), 2, 3, 5 (interf + soc act), 6, 7 | 1, 4, 5, 6, 7 | 2, 3, 7 | 2, 3 (only items 2, 3, 6, 7 applicable for analysis) | 1, 2, 4, 5, 6, 7 |
|  |  |  |  |  |  |  |
| 20 | pre-post | 1 (walk), 2, 6 | 4, 5, 6 | 5 | 4, 6 (only items 1, 4, 6, 7 applicable for analysis) | 5 |
|  |  |  |  |  |  |  |
|  | post-then | 1 (trouble), 2, 3, 6, 7 | 1, 2, 3, 4, 5, 6, 7 | 5, 7 | 4, 7 (only items 1, 4, 6, 7 applicable for analysis) | 6 |
|  |  |  |  |  |  |  |

| 21 | pre-post | 1, 3, 4, 5, 7 | 1, 3, 5, 7 | 7 | 4 (only items 4, 7 applicable for analysis) | 1, 3, 4, 7 |
| --- | --- | --- | --- | --- | --- | --- |
|  |  |  |  |  |  |  |
|  | post-then | 2, 3, 4, 5 (interference + soc activities) | 2, 3, 5, 7 | 7 | 7 (only items 4, 7 applicable for analysis) | 3, 4, 5 |
|  |  |  |  |  |  |  |
| 22 | pre-post | 4, 5, 6, 7 | 1, 3, 6 | 6 | (no items applicable for analysis) | 4, 6, 7 |
|  |  |  |  |  |  |  |
|  | post-then | 3, 5 (interference), 6, 7 | 1, 6 | 1, 4, 7 | (no items applicable for analysis) | 2, 4, 6, 7 |
|  |  |  |  |  |  |  |
| 23 | pre-post | 1 (trouble), 2, 5 (interference), 6, 7 | 1, 2, 3, 4, 5, 6, 7 | 2, 3, 4, 5, 6, 7 | 4, 7 (only items 4, 7 applicable for analysis) | 3, 4, 6 |
|  |  |  |  |  |  |  |
|  | post-then | 1 (trouble), 2, 3, 5 (interference), 6, 7 | 1, 4, 5, 6, 7 | 2, 5, 6, 7 | 4 (only items 4, 7 applicable for analysis) | 3 |
|  |  |  |  |  |  |  |
| 24 | pre-post | 1 (trouble + walk), 2, 3, 5 (interference), 6, 7 | 2, 3, 5, 6, 7 | 6 | (no items applicable for analysis) | 4 |
|  |  |  |  |  |  |  |
|  | post-then | 1 (trouble), 2, 3, 5 (interf + soc act), 6, 7 | 3, 5, 6, 7 | 3 | (no items applicable for analysis) | 7 |
|  |  |  |  |  |  |  |

* Item 1: Do you have any trouble taking a short walk outside of the house?; Item 2: Have you had pain?; Item 3: Were you tired?; Item 4: Did you worry?; Item 5: Has your physical condition or medical treatment interfered with your social activities?; Item 6: How would you rate your overall health during the past week?; Item 7: How would you rate you overall quality of life during the past week?

** Analysis only possible when respondents used combinatory algorithm at both pretest and posttest or posttest and thentest.

**Supplementary Table 2: Assumption 2: Results at the individual patient level, with the numbers reflecting the item numbers***

| Patients | Time frame: similar Description: similar | Time frame: similar  Description: dissimilar | Time frame: dissimilar Description: similar | Time frame: dissimilar Description: dissimilar |
| --- | --- | --- | --- | --- |
|  |  |  |  |  |
| 1 | 2, 3, 4, 5 |  | 6 | 1, 7 |
| 2 | 6 | 2, 3, 4, 7 |  | 1, 5 |
| 3 | 1, 3, 5, 7 | 6 |  | 2, 4 |
| 4 | 1, 2, 4, 6 | 7 |  | 3, 5 |
| 5 | 1, 2, 4 | 5 |  | 3, 6, 7 |
| 6 | 1, 2, 3, 4, 6, 7 |  |  |  |
| 7 | 1, 2, 4, 5, 6 | 3, 7 |  |  |
| 8 | 2, 4, 5 | 7 |  | 1, 3, 6 |
| 9 | 7 | 1, 2, 5, 6 |  | 3, 4 |
| 10 | 4, 7 | 1, 3 | 5, 6 | 2 |
| 11 | 2, 4 | 5, 6 |  | 7 |
| 12 | 2, 3 |  |  | 1, 4, 5, 6, 7 |
| 13 | 6, 7 | 1, 5 |  | 2, 3, 4 |
| 14 |  | 3, 6 |  | 1, 2, 4, 5, 7 |
| 15 | 3, 4, 7 | 2, 5 | 1, 6 |  |
| 16 | 1, 3, 4, 5 |  |  | 2 |
| 17 | 1, 2, 3 | 7 |  | 4, 5, 6 |
| 18 | 5, 6, 7 |  | 1, 3 | 2, 4 |
| 19 | 5 | 3, 7 |  | 1, 2, 4, 6 |
| 20 | 4, 6 |  | 5 | 1, 2, 3, 7 |
| 21 | 2 | 4, 7 |  | 1, 3, 5 |
| 22 | 2, 7 | 4, 5 | 6 | 1, 3 |
| 23 |  | 5 | 1 | 2, 3, 4, 6, 7 |
| 24 | 3, 5 | 1, 4,7 |  | 2, 6 |

* Item 1: Do you have any trouble taking a short walk outside of the house?; Item 2: Have you had pain?; Item 3: Were you tired?; Item 4: Did you worry?; Item 5: Has your physical condition or medical treatment interfered with your social activities?; Item 6: How would you rate your overall health during the past week?; Item 7: How would you rate you overall quality of life during the past week?
